# Klippel–Trénaunay Syndrome With Pelvic/Rectal and Urinary Bladder Involvement: A Rare Case Report

**DOI:** 10.1002/ccr3.73567

**Published:** 2026-09-28

**Authors:** Somaya Al Kiswani, Khitam Salahat, Omar Daas, Mohammed AbdulJabbar Abed, Reem Abuhamdah, Usama Al Khuffash, Abdullah Nofal

**Affiliations:** ^1^ Radiology Department King Hussein Cancer Center Amman Jordan; ^2^ Department of Medicine, Faculty of Medicine and Health Sciences An‐Najah National University Nablus Palestine; ^3^ Department of Medicine, Faculty of Medicine The University of Jordan Amman Jordan

**Keywords:** arteriovenous malformation, gastrointestinal hemorrhage, hematuria, Klippel–Trénaunay syndrome, multidisciplinary care, vascular malformations

## Abstract

Concurrent bladder and rectosigmoid vascular malformations in Klippel–Trénaunay syndrome can mimic synchronous pelvic malignancy. Multidisciplinary clinical, radiological, endoscopic, and pathological evaluation is essential to avoid misdiagnosis and to guide appropriate, often conservative, management.

## 1 Introduction

Klippel–Trénaunay syndrome (KTS), also known as Klippel–Trénaunay–Weber syndrome, is a vascular malformation condition characterized by varying involvement of cutaneous capillaries, veins, and lymphatics, as well as hypertrophy of the affected limb's soft tissues and bones [1]. KTS can manifest at birth, early infancy, or childhood. The incidence estimates range from 2 to 5 cases per 100,000 people. Males are more commonly affected than females, and no racial preference has been found [2]. Visceral involvement is rare, but it can be a major cause of morbidity and mortality. Organs that may be affected include the bladder, rectum, gastrointestinal tract, penis, uterus, vulva, vagina, liver, kidneys, lungs, and spine. In 18% of patients, pelvic involvement manifests as rectal bleeding or hematuria; in 12.5% of cases the rectum and distal colon are affected [3]. Medical imaging is essential for diagnosing KTS, evaluating its severity and complications, monitoring it, and distinguishing it from other illnesses of a similar nature [4]. Imaging starts with radiographs and sonography, but computed tomography (CT) is better at displaying bone abnormalities and phleboliths. Furthermore, magnetic resonance imaging (MRI) angiography is now replacing contrast angiography [5].

KTS is primarily treated conservatively, and patients typically need to be followed for the rest of their lives. The primary goals of treatment are to manage any bleeding episodes, relieve symptoms, and prevent and treat consequences such as cellulitis, deep vein thrombosis, chronic coagulopathy, and congestive heart failure [6].

We report a rare case of KTS presenting with hematuria and bleeding per rectum in a young adult with two separate masses.

## 2 Case History/Examination

A 37‐year‐old Yemeni man, an ex‐smoker, married, with no significant past medical or surgical history, presented with gross hematuria with clots, rectal bleeding, lower‐back pain radiating to the right leg, and lower abdominal pain. His initial hemoglobin, measured at another hospital in late 2023, was 6 g/dL. Two months before evaluation at our center, he underwent cystoscopy and colonoscopy, which—according to the patient—revealed a bladder mass, but no report of the aforementioned procedures was available to us.

The patient was referred to our center in February 2024. He had received blood transfusions and his hemoglobin at referral was 12.4 g/dL (post‐transfusion); INR was 1.23, creatinine 0.7 mg/dL, and platelet count 197 × 10^9^/L. Physical examination showed no port‐wine stains, no cutaneous capillary malformations, and no lower limb hypertrophy or varicose veins.

## 3 Differential Diagnosis, Investigations and Treatment

He continued to have rectal bleeding and gross hematuria with clots. CT of the chest, abdomen, and pelvis revealed a fundal bladder‐wall mass with features suggestive of malignancy, measuring approximately 5.3 × 5.4 × 4.8 cm, and circumferential rectal wall thickening. These findings initially raised concern for pelvic malignancy (Figure 1).

**FIGURE 1:**
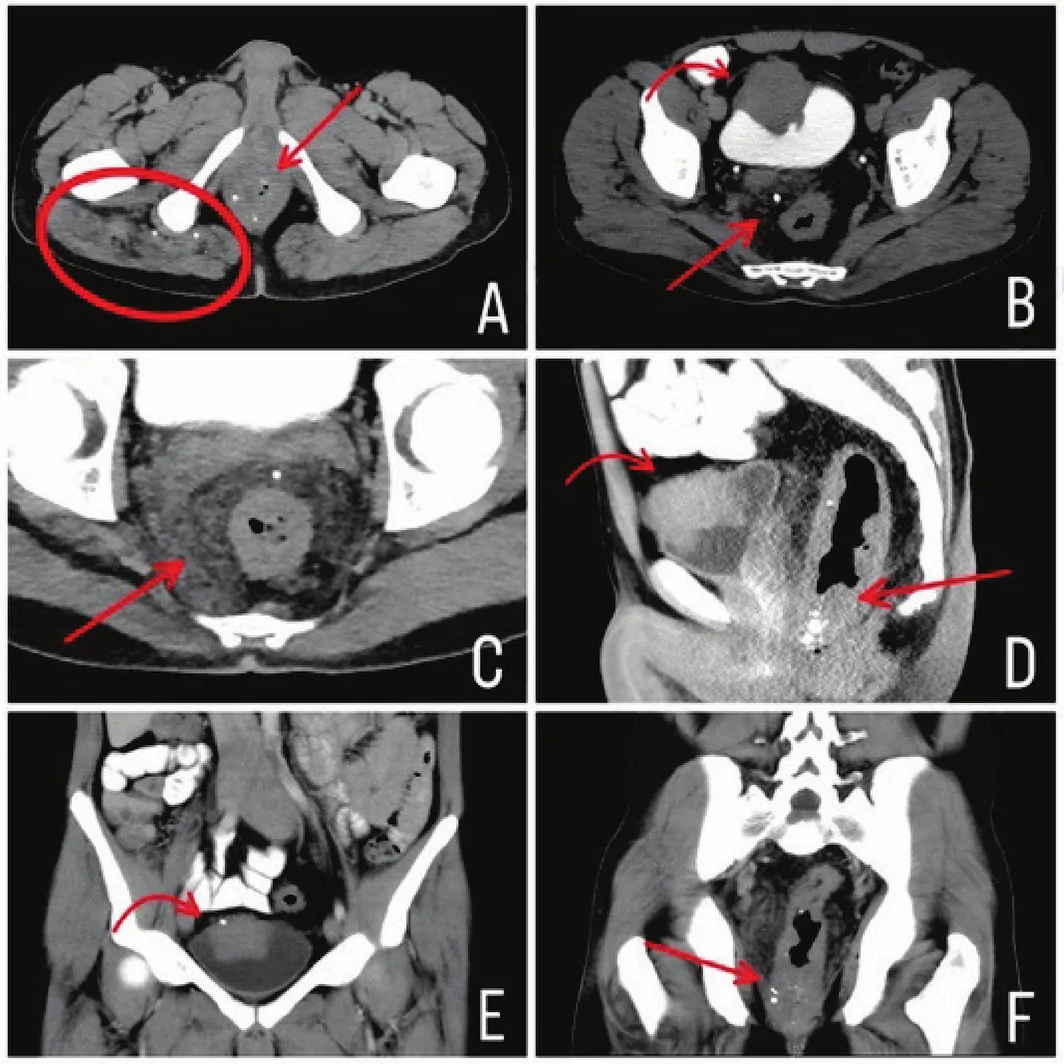
Contrast‐enhanced CT images of the abdomen and pelvis demonstrating extensive pelvic vascular malformation changes. (A) Axial non‐contrast CT image demonstrating circumferential rectal wall thickening with multiple associated phleboliths (long arrow). Rounded annotation highlights asymmetric enlargement/fatty infiltration involving the right gluteus maximus muscle. (B) Delayed axial contrast‐enhanced CT image demonstrating the anterior urinary bladder wall mass (curved arrow). (C) Magnified delayed axial contrast‐enhanced CT image demonstrating mesorectal/perirectal fat stranding with obscuration of fat planes and adjacent phleboliths. (D) Sagittal contrast‐enhanced CT image demonstrating irregular rectal wall thickening with associated pelvic phleboliths (straight arrow) and the anterior urinary bladder wall mass lesion (curved arrow). (E) Coronal venous‐phase contrast‐enhanced CT image demonstrating the anterior urinary bladder wall mass (curved arrow). A straight arrow demonstrates associated rectal wall thickening and pelvic phleboliths. (F) Coronal venous‐phase contrast‐enhanced CT image demonstrating circumferential rectal wall thickening with multiple associated pelvic phleboliths and surrounding mesorectal/perirectal fat stranding.

Subsequent MRI of the abdomen and pelvis with and without contrast demonstrated a 5 cm irregular anterior bladder‐wall mass with massive infiltration of the perivesical fat, severe mucosal thickening, and submucosal oedema of the rectosigmoid. No significant pelvic lymphadenopathy, ascites, or destructive osseous lesions were seen (Figures 2 and 3).

**FIGURE 2:**
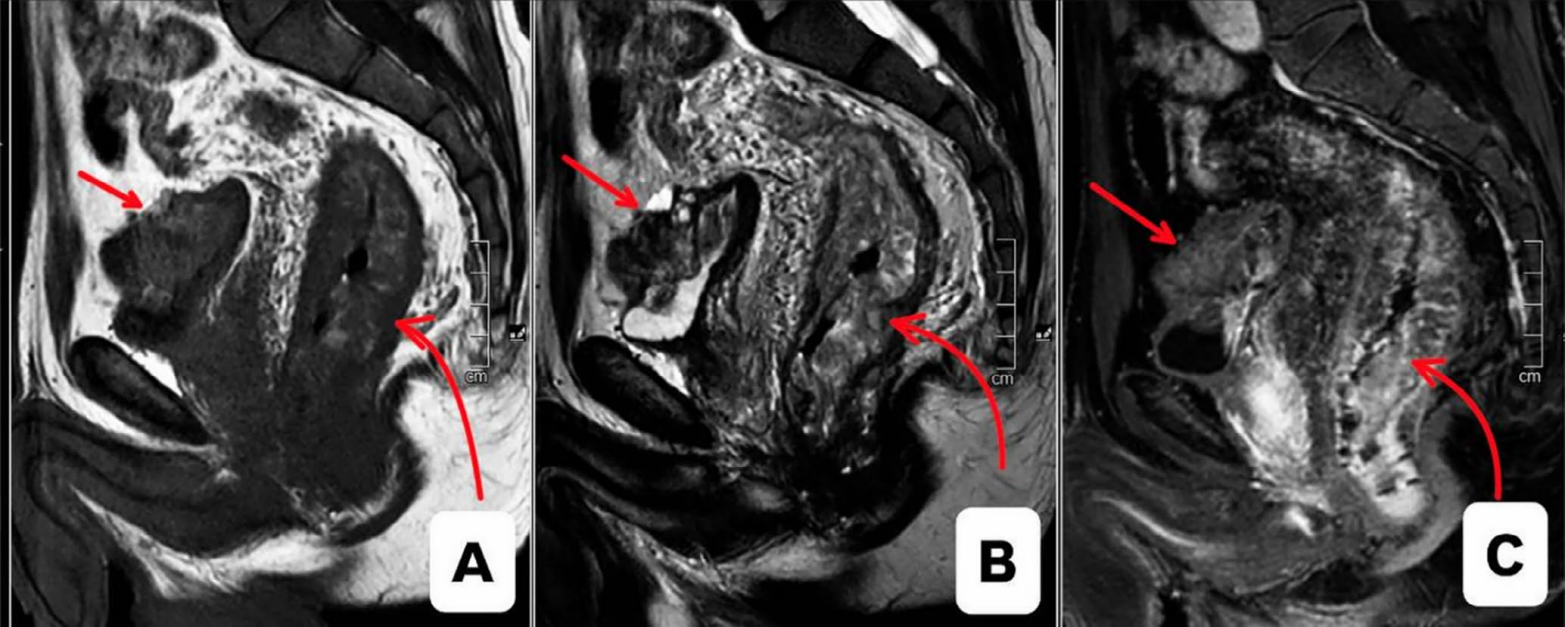
MRI pelvis demonstrating complex pelvic vascular malformation involving the urinary bladder and rectum. (A) Sagittal T1‐weighted MRI image demonstrating irregular anterior urinary bladder wall mass lesion (short arrow) with associated rectal wall thickening (curved arrow). (B) Sagittal T2‐weighted MRI image demonstrating marked rectosigmoid wall thickening and heterogeneous pelvic soft tissue signal abnormalities (curved arrow), with persistent anterior urinary bladder wall lesion (short arrow). (C) Sagittal post‐contrast T1‐weighted MRI image demonstrating heterogeneous enhancement of the anterior urinary bladder wall mass (short arrow) and associated enhancing rectal wall thickening/adjacent vascular soft tissue abnormality (curved arrow).

**FIGURE 3:**
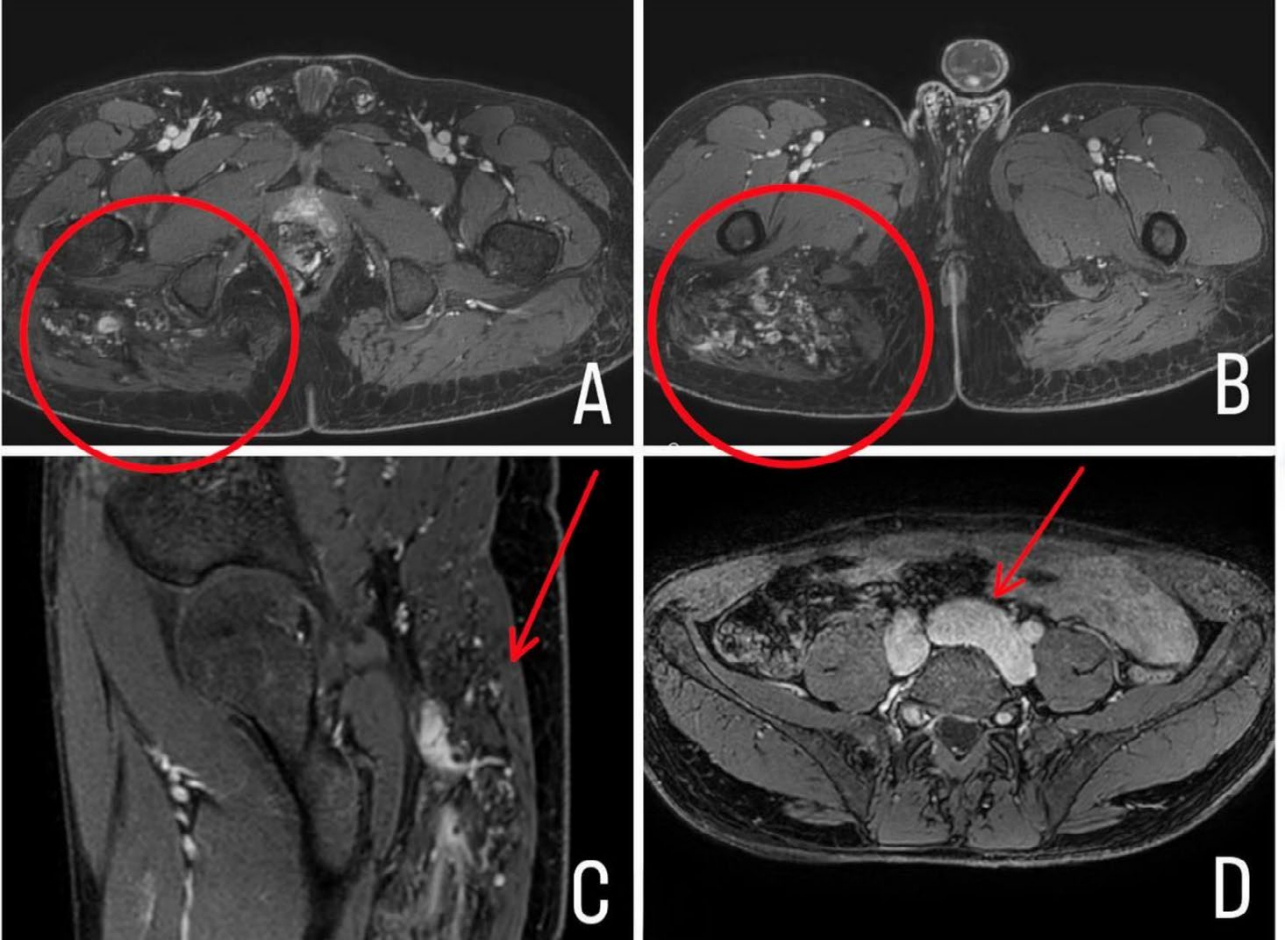
Post‐contrast fat‐suppressed T1‐weighted MRI images demonstrating additional findings supporting an underlying complex vascular malformation syndrome within the Klippel–Trénaunay spectrum. (A) Axial MRI image demonstrating asymmetric abnormal appearance of the right gluteal region with heterogeneous fatty infiltration/chronic fatty replacement of the right gluteus maximus muscle (circles). (B) Axial MRI image demonstrating prominent enhancing vascular channels within the abnormal right gluteal region (straight arrow). (C) Sagittal MRI image demonstrating enlarged vascular channels extending through the right gluteal and pelvic regions (curved arrow). (D) Axial MRI image demonstrating enlarged bilateral iliac venous structures, more prominent on the left side (straight arrows).

Given the imaging appearance of an infiltrative bladder‐wall mass with circumferential rectal wall thickening, the initial differential diagnosis was broad and included both malignant and non‐malignant etiologies. Primary pelvic malignancy such as a urothelial bladder carcinoma or a rectosigmoid carcinoma with local extension was considered the leading concern given the size, infiltrative margins, and perivesical fat involvement of the lesion. However, the presence of multiple phleboliths, diffusely enhancing tortuous vascular channels, and the absence of restricted diffusion, significant lymphadenopathy, or destructive osseous lesions raised the possibility of an underlying vascular malformation. Additional considerations included an isolated non‐syndromic arteriovenous malformation, a bladder or rectal hemangioma, inflammatory bowel disease (given the hyperemic, vascularized mucosa on colonoscopy), a hemorrhoidal or other anorectal bleeding source, colonic angiodysplasia, and a lymphatic malformation as part of a mixed vascular lesion. These possibilities, along with the features that supported or excluded each, are summarized in Table 1. Tissue diagnosis was therefore required to differentiate malignancy from a vascular malformation and to guide subsequent management.

**TABLE 1:** Differential diagnosis considered for the pelvic bladder and rectal masses.

Differential diagnosis	Distinguishing features	Relevance/Exclusion in this patient
Pelvic malignancy (urinary bladder or rectal/rectosigmoid carcinoma)	Discrete, often irregular soft‐tissue mass; may show restricted diffusion, regional lymphadenopathy, and local tissue invasion	Initially favored given the size and infiltrative appearance of the bladder mass on CT/MRI; excluded by benign histopathology on TURBT and on the final resection specimen, with no malignant cells identified
KTS‐associated visceral vascular malformation	Diffuse, infiltrative, poorly marginated lesion with phleboliths, enhancing tortuous vascular channels, and lack of a discrete capsule	Ultimately confirmed; supported by phleboliths on CT, enhancing vascular channels on MRI, hyperemic/vascularized mucosa on colonoscopy, and final histopathology showing mixed vascular channels
Isolated (non‐syndromic) arteriovenous malformation	Vascular channels without associated cutaneous, limb, or multi‐organ findings	Considered but felt less likely; the multi‐site pattern (rectal, bladder, and right gluteal involvement) favored a syndromic vascular malformation within the KTS spectrum rather than a sporadic AVM
Hemangioma of the bladder or rectum	Well‐circumscribed vascular lesion, may contain phleboliths, typically less infiltrative	Considered given phlebolith formation; distinguished by the diffuse, multifocal, and infiltrative extent of the lesion, atypical for a simple hemangioma
Inflammatory bowel disease (e.g., ulcerative colitis, Crohn disease)	Mucosal inflammation and ulceration, wall thickening, minimal mass effect	Considered because of hyperemic mucosa on colonoscopy; excluded by absence of a typical ulcerative/inflammatory mucosal pattern and by histopathology confirming a vascular malformation rather than inflammatory changes
Hemorrhoidal or other anorectal source of bleeding	Bleeding from anal cushions or superficial anorectal vessels; no pelvic mass or bladder involvement	Excluded given the presence of a pelvic/bladder mass and rectal wall thickening on cross‐sectional imaging, well beyond an anorectal source
Colonic angiodysplasia	Small, flat mucosal or submucosal vascular lesions, usually without an associated mass	Excluded given the size of the lesion and concurrent bladder involvement, both atypical for isolated angiodysplasia
Lymphatic malformation	Cystic, non‐enhancing or minimally enhancing spaces on MRI	Considered as a component of a mixed malformation; confirmed on final histopathology, which demonstrated lymphatic spaces alongside venous, arterial, and cavernous vascular channels

In March 2024, the patient underwent transurethral resection of bladder tumor (TURBT). A large, irregular mass arising from the bladder dome was identified; histopathology revealed cauterized fragments of muscularis mucosae and muscularis propria with focally denuded urothelial lining. No definite malignancy was found.

Despite the benign histology, the patient continued to experience rectal bleeding, lower‐back pain radiating to the right leg, and diarrhea. He underwent several interventional radiology procedures, including embolization. In August 2024, a multidisciplinary review concluded that the bladder lesion was most likely vascular rather than malignant. Colonoscopy later revealed inflamed, hyperemic mucosa, extremely vascularized mucosa, and diffusely dilated superficial veins—findings consistent with the cystoscopic appearance.

By October 2024, he had developed laboratory evidence of chronic consumptive coagulopathy: PT 17.1 s (normal 11.7–15.2), APTT 41.6 s (28.1–38.1), INR 1.30 (0.87–1.15).

Due to refractory pelvic discomfort and continuous bleeding, he required repeated radiological treatments and pelvic angioembolization procedures, leading to multiple blood transfusions and escalating analgesic requirements.

In November 2024, he underwent laparoscopic low anterior resection with a diverting loop ileostomy. Key intraoperative findings included vascular malformations surrounding the rectum and a hard mass in the upper rectum that appeared to arise from the bladder. A colonic anastomosis was created; no hepatic or peritoneal metastases were present, and the air‐leak test was negative. The inter‐mesenteric plane was used to prevent excessive bleeding from the vascular malformations.

Final histopathology from the resected specimen demonstrated an arteriovenous malformation with lymphovascular malformation features, mixed vascular channels (veins, arteries, cavernous spaces, and lymphatic spaces), fibro‐intimal thickening, surface erosion, fat necrosis, a foreign‐body giant cell reaction, thrombus formation, and reactive lymph nodes. No malignancy was identified. These findings were consistent with a complex vascular malformation syndrome within the Klippel–Trénaunay spectrum.

On 25 November 2024, a presacral collection measuring 6.6 × 3.3 cm was detected. The patient underwent CT‐guided drainage and received intravenous piperacillin‐tazobactam and vancomycin. He was discharged after resolution of the collection.

## 4 Outcome and Follow‐Up

He underwent ileostomy closure in February 2025. However, he continued to have perianal pain of stabbing/stretching quality and incontinence, requiring ongoing pain management and bleomycin‐based sclerotherapy for the residual vascular mass. A follow‐up pelvic MRI in March 2026 showed persistent arteriovenous malformations involving the anterior bladder wall, right pelvic side wall, and right sciatic foramen, with extensive involvement of the right gluteal muscles.

## 5 Discussion

KTS is a clinical diagnosis based on at least two of the three typical findings: localized cutaneous capillary malformations, venous abnormalities, and limb hypertrophy. The degree of the overgrowth segment and the severity of the component vascular distortion have a significant prognostic impact. Although the syndrome often has a benign course, problems arising from its different components may lead to serious systemic complications, including vascular, hematological, and neurological sequelae [7].

Gastrointestinal involvement accounts for approximately 20% of KTS cases. The most commonly affected areas are the rectum and sigmoid colon. But the entire bowel may also be affected. The most common area impacted in our patient was the rectum, consistent with previous reports identifying the rectum and sigmoid colon as the most frequently involved bowel segments in KTS. Clinical symptoms vary from symptom‐free to abdominal pain and mild to severe bleeding. Hematochezia was among the most frequent presenting symptoms. Rectal bleeding results from internal iliac vein overflow secondary to deep vein agenesis in the lower limb [8]. Endoscopic examination should be used to investigate GI bleeding in a patient with suspected KTS. Although endoscopy has the benefit of demonstrating the location and extent of vascular malformations, it may be misleading to attribute bleeding to the vascular malformations seen during the endoscopy because of potential multifocal vascular malformations in the various GI tract regions, so endoscopic examination of the entire GI tract should be performed as normal clinical practice for the precise location and appropriate therapy of GI bleeding in patients with KTS [7, 9]. Despite the discrepancy between histopathological and radiological results, colonoscopy revealed findings that support the diagnosis.

KTS can also affect the genitourinary tract; it accounts for 10% of cases and manifests as hydronephrosis, hematuria, and trouble voiding [10]. The urethra, ureter, vulva, penis, and bladder may be affected. Other rare pelvic manifestations, including uterine involvement, have also been reported in KTS [11]. Although simultaneous gastrointestinal and genitourinary involvement is uncommon in KTS [7], our patient also presented with hematuria. Investigations revealed a bladder mass separate from the mass in the rectum. According to previous studies, GU involvement mostly relates to bleeding from urethral and bladder vascular abnormalities, caused by internal iliac vein reflux and rupture of the vesical venous plexus [12]. Confirmed cases of hematuria originating from the kidney or ureter are rarely documented; endoscopic and angiographic methods can alleviate the majority of KTS hematuria [12]. Currently, endoscopic lasers are thought to have a deep thermal effect and beneficial tissue coagulation effect, making them an effective approach for treating bladder hemangiomatous lesions [2]. Our patient underwent several angioembolization therapies to control bleeding.

There is no curative treatment for patients with KTS, and treatment is predominantly conservative and symptomatic, aimed primarily at improving the patient's quality of life.

The effectiveness of surgery depends on the preoperative evaluation of the deep venous system utilizing techniques such as computed tomography (CT) arteriography and duplex scanning contrast venography, which can assist in ascertaining the extent of vascular involvement and the presence of arteriovenous fistulae [2]. Our patient underwent rectal mass resection due to persistent bleeding and a drop in hemoglobin.

This case demonstrates the diagnostic challenges posed by visceral KTS; coexisting bladder and rectosigmoid involvement can be misinterpreted as invasive pelvic cancer. A multidisciplinary approach integrating clinical, radiological, endoscopic, surgical, and pathological findings is required for accurate diagnosis and management.

## Author Contributions

**Somaya Al Kiswani:** conceptualization, supervision, writing – review and editing. **Mohammed AbdulJabbar Abed:** visualization. **Reem Abuhamdah:** writing – review and editing. **Usama Al Khuffash:** writing – review and editing. **Abdullah Nofal:** supervision, writing – review and editing. **Omar Daas:** data curation. **Khitam Salahat:** writing – original draft.

## Funding

The authors have nothing to report.

## Disclosure

Guarantor: Somaya Al Kiswani, MD, accepts full responsibility for the integrity of the work, had access to the data, and controlled the decision to publish.

## Ethics Statement

Our institution does not require ethical approval/waiver for case reports.

## Consent

Written informed consent was obtained from the patient's family for publication of this case report and any accompanying images.

## Conflicts of Interest

The authors declare no conflicts of interest.

## Data Availability

The data supporting the findings of this case report are contained within the article. Additional details are available from the corresponding author upon reasonable request.
